# Temporal precision in population—but not individual neuron—dynamics reveals rapid experience-dependent plasticity in the rat barrel cortex

**DOI:** 10.3389/fncom.2014.00155

**Published:** 2014-11-25

**Authors:** Seif Eldawlatly, Karim G. Oweiss

**Affiliations:** ^1^Department of Computer and Systems Engineering, Faculty of Engineering, Ain Shams UniversityCairo, Egypt; ^2^Department of Electrical and Computer Engineering, University of FloridaGainesville, FL, USA; ^3^Department of Biomedical Engineering, University of FloridaGainesville, FL, USA; ^4^Department of Neuroscience, University of FloridaGainesville, FL, USA; ^5^Department of Electrical and Computer Engineering, Michigan State UniversityEast Lansing, MI, USA

**Keywords:** effective connectivity, whisker pairing, barrel cortex, experience-dependent plasticity, Dynamic Bayesian Network

## Abstract

Cortical reorganization following sensory deprivation is characterized by alterations in the connectivity between neurons encoding spared and deprived cortical inputs. The extent to which this alteration depends on Spike Timing Dependent Plasticity (STDP), however, is largely unknown. We quantified changes in the functional connectivity between layer V neurons in the vibrissal primary somatosensory cortex (vSI) (barrel cortex) of rats following sensory deprivation. One week after chronic implantation of a microelectrode array in vSI, sensory-evoked activity resulting from mechanical deflections of individual whiskers was recorded (control data) after which two whiskers on the contralateral side were paired by sparing them while trimming all other whiskers on the rat's mystacial pad. The rats' environment was then enriched by placing novel objects in the cages to encourage exploratory behavior with the spared whiskers. Sensory-evoked activity in response to individual stimulation of spared whiskers and adjacent re-grown whiskers was then recorded under anesthesia 1–2 days and 6–7 days post-trimming (plasticity data). We analyzed spike trains within 100 ms of stimulus onset and confirmed previously published reports documenting changes in receptive field sizes in the spared whisker barrels. We analyzed the same data using Dynamic Bayesian Networks (DBNs) to infer the functional connectivity between the recorded neurons. We found that DBNs inferred from population responses to stimulation of each of the spared whiskers exhibited graded increase in similarity that was proportional to the pairing duration. A significant early increase in network similarity in the spared-whisker barrels was detected 1–2 days post pairing, but not when single neuron responses were examined during the same period. These results suggest that rapid reorganization of cortical neurons following sensory deprivation may be mediated by an STDP mechanism.

## Introduction

The brain develops and adapts through multifaceted plastic changes in connectivity among its neurons (Siegelbaum and Kandel, 1991; Dan and Poo, 2004). Experience-dependent plasticity might be the most vital, as it critically affects the organism's ability to compensate for sensory alteration (Fox and Wong, 2005). For example, amputating hand fingers in primates, or blocking nerves that innervate them results in reorganization of the primary somatosensory cortex (SI) (Kaas, 1991; Weiss et al., 2000), although recent data suggest this re-organization may be caused by plasticity in other subcortical areas (Kambi et al., 2014). In rodents, the primary somatosensory cortex is required for whisker-based tactile sensation of objects in the surrounding (Guo et al., 2014; Petersen, 2014). The interaction between cortical inputs caused by self-generated whisker movements and inputs from whisker-object contact seems to be critical for rapid decision making about when and what is being touched. Anatomically, the vibrissal area of SI (vSI) is innervated by numerous afferents from whisker follicles on the mystacial pad, and has been shown to extensively reorganize following *whisker pairing*—the process of depriving the rat from all but two adjacent whiskers (Feldman and Brecht, 2005). In particular, neuronal firing rates in one principal spared-whisker (W1) barrel have been shown to resemble those in the other spared-whisker (W2) barrel in response to deflection of W2 (Diamond et al., 1993). This may be attributed to the emergence of new connections between the spared-whisker barrels or to the strengthening of already existing connections (Hebb, 1949; Lebedev et al., 2000; Feldman and Brecht, 2005). The precise mechanism that may mediate this re-organization at the population level, however, remains largely unknown.

Tracking experience-dependent plasticity *in vivo* is challenging for a number of reasons. Chief among all is the multifaceted aspects of plasticity that may occur at an individual synapse, a single cell, or a population level and at multiple time scales, which are currently insurmountable to measure in the intact brain of an awake behaving animal. As such, characterizing experience-dependent plasticity has been limited to the analysis of *individual* unit response. Changes in spike count and spike timing have been mostly used as biomarkers of experience-dependent plasticity (Celikel et al., 2004; Jacob et al., 2007). On the other hand, population analysis has been primarily limited to the analysis of *pairwise* correlations (Lebedev et al., 2000; Erchova and Diamond, 2004; Marre et al., 2009). More recently, graph-based techniques were shown to be more effective in characterizing stimulus encoding properties in the rat barrel cortex (Eldawlatly and Oweiss, 2011; Adibi et al., 2014), in retinae (Pillow et al., 2008; Ganmor et al., 2011) as well as in other cortices (Tang et al., 2008).

Here we sought to characterize the dynamics of neural ensembles in rat vSI to test the hypothesis that rapid cortical re-organization following sensory deprivation induced by whisker pairing may be mediated, in part, by a Spike Timing Dependent Plasticity (STDP) mechanism. Using a combination of chronic ensemble recording of multiple single unit activity *in vivo* and the statistical inference power of Dynamic Bayesian Networks (DBN)—a class of graph-based methods—we demonstrate that whisker pairing results in significant changes in DBN-derived effective connectivity between locally recorded populations of layer V vSI neurons. Given the sensitivity of DBNs to the temporally ordered spike times in the recorded ensemble spike patterns, these changes could be explained in part by an STDP mechanism.

## Materials and methods

### Barrel cortex recording and electrode implantation

Four adult female Sprague Dawley rats weighing ~300 g were used in this study. All procedures involving animals were approved by the Michigan State University Institutional Animal Care and Use Committee (IACUC). Animals were anesthetized using a cocktail of ketamine and xylazine (75 and 5 mg/kg injected intrapertoneally, respectively). The left somatosensory cortex was exposed (4 × 4 mm craniotomy, 0–4 mm posterior and 4–8 mm lateral to bregma). A 32-channel microelectrode silicon array (NeuroNexus Technologies, Ann Arbor, MI, USA) with 8 shanks, 4 recording sites/shank, 200 μm shank separation and 100 μm electrode separation within shank was advanced into the barrel field in 100 μm steps. Acquired signals were amplified and band-pass filtered in the range 300–5000 Hz and sampled at 25 KHz (Tucker-Davis Technologies, Alachua, FL, USA). Stimulus-driven activity was observed at depths of 1100–1500 μm corresponding to layer V of the barrel cortex. After reaching the desired depth in layer V, the electrode array was secured in place using dental cement.

Rats were left to recover for 7–10 days post-surgery after which they were anesthetized and stimulus-driven S1 activity was recorded (control data). For each rat, 3 whiskers contralateral to the side of the implant were selected for mechanical stimulation based on the observed neuronal response to manual deflection (mapping experiment). The selected whiskers were deflected individually by inserting each whisker into a capillary tube glued to a piezoelectric bimorph (Piezo Systems, Cambridge, MA, USA) where the distance between the tube and the skin was kept at ~1 mm. As such, a whisker, whether spared or re-grown, was always stimulated at an identical distance from the follicle and therefore is not expected to drive a different input to the cortex than what was intended to be delivered. Each whisker was horizontally deflected 900 times with a displacement of 80 μm for 100 ms (rise time and fall time were each set to 1 ms) at 1 Hz frequency (i.e., the inter-trial interval was 900 ms). Two whiskers were then selected to be paired and all other whiskers on the same side of the rat's mystacial pad were trimmed to the skin level. Rats were returned back to their cages where enrichments were introduced to encourage them to actively whisk new objects using the spared whiskers. After 1–2 days and 6–7 days post-whisker trimming, rats were re-anesthetized and stimulus-driven activity was recorded (plasticity data) in response to spared whiskers deflection as well as one re-grown adjacent whisker. At the end of the 1–2 days recording session, re-grown whiskers (not previously spared) were trimmed again to restrict the pairing to the same pair of whiskers.

For both control and plasticity data, spikes from multiple single unit activity were detected and sorted using NeuroQuest; a MATLAB toolbox for neural data processing and analysis (Kwon et al., 2012). Spikes were declared present if the raw waveform surpassed a threshold set at 3 times the noise standard deviation. A spike length of ~1 ms was used for spike sorting (0.25 ms pre threshold crossing and 0.75 ms post threshold crossing). Spikes were aligned at their trough. Principal Component Analysis (PCA) was applied to the detected spikes, and the first 2 principal components were used as features for spike sorting. An average population size of 23.2 ± 6.7 single units/rat was recorded. Spike trains were binned at Δ = 1 ms.

### Dynamic Bayesian Networks (DBNs)

A graphical model uses a *node* to represent a random variable and an *edge* to connect two nodes representing a probabilistic relationship between the corresponding random variables. Formally, a graph *G* consists of a set of nodes *V* and edges *E*, written as *G* = <*V, E*>. Each node in *V*, denoted by *v_i_*, corresponds to a random variable *x_i_*. Each edge in *E* ⊆ *V* × *V*, denoted by *v_i_* → *v_j_*, indicates the causal influence of random variable *x_i_* on random variable *x_j_* (i.e., effective connectivity). Graphical representation of neuronal interactions is intuitive because it can be easily visualized and allows the use of many established graph metrics to quantify certain features of the inferred graph that can be compared to imaging and anatomical neural data (Stam and Reijneveld, 2007; Bullmore and Sporns, 2009).

In the DBN analysis we carried out on the spike train data, a directed acyclic graph (DAG) (Murphy, 2002), denoted by *G*, and a set of conditional probabilities, denoted by *P*, represented the statistical dependence between the simultaneously observed spike trains (*r*_1_, *r*_2_, …, *r_n_*), and was used to represent the network *B* as *B* = <*G, P*>. Each node in *V*, denoted by *v_i_*, corresponds to the spike train of neuron *i* at time *t*, where *r_i_*(*t*) = 1 represents a “spike,” and *r_i_*(*t*) = 0 represents “no spike.” Each directed edge in *E* indicates conditional dependence (i.e., causal influence) among the corresponding neurons.

The state of each variable *r_i_*(*t*) in the DBN is determined only by its putative pre-synaptic cells' history, denoted **r**_π(*i*)_(1 : *t* − 1), and is independent of the state of any other cell. Thus, the probability Pr (*r*_1_(*t*), *r*_2_(*t*), …, *r_n_*(*t*)|**r**(1 : *t* − 1)) can be expressed in terms of the conditional probabilities Pr (*r_i_*(*t*)|**r**_π(*i*)_(1 : *t* − 1)) as

(1) $$\begin{array}{l} \mathrm{Pr}\left(r_{1}(t),r_{2}(t),\ldots ,r_{n}(t)|r(1:t-1)\right)\\ \;=\prod_{i=1}^{n}\mathrm{Pr}\left(r_{i}(t)|r_{\pi (i)}(1:t-1)\right). \end{array}$$

For the sake of simplicity, it is often assumed that *r_i_*(*t*) is only dependent on the value of its parents observed at time *T* = *t* − 1, which simplifies the conditional probabilities Pr (*r_i_*(*t*)|**r**_π(*i*)_(1 : *t* − 1)) to Pr (*r_i_*(*t*)|**r**_π(*i*)_(*t* − 1)). This is known as the Markov assumption with Markov lag equal to 1. This simplification to the Markov assumption can be extended to include multiple Markov lags. For instance, a DBN with maximum Markov lag equals 3 implies that *r_i_*(*t*) is decided by the value of its parents observed at time *T* = *t* − 1, *t* − 2, *t* − 3, or Pr (*r_i_*(*t*)|**r**_π(*i*)_(*t* − 3: *t* − 1)).

Learning DBN structure (i.e., inferring the edges in the network) from the data can be achieved by searching for the structure G^*^ that maximizes the posterior density of the network structure *G* for a given dataset *D*, denoted Pr(*G*|*D*), expressed using Bayes' rule as

(2) $$\mathrm{Pr}(G|D)=\frac{\mathrm{Pr}(D|G)\mathrm{Pr}(G)}{\mathrm{Pr}(D)}$$

where Pr(*D*|*G*) is the likelihood of the data *D* given the structure *G*, Pr(*G*) is the structure prior, and Pr(*D*) is the probability of the observed data. Assuming no prior information about the structures [i.e., a uniform distribution for Pr(*G*)] and given that Pr(*D*) is independent of the choice of *G*, G^*^ can be found as the structure that maximizes Pr(*D*|*G*).

Score-based approaches can be used to search for G^*^ in which multiple network structures are evaluated by assigning a score to Pr(*D*|*G*) (Heckerman, 1995). In our analysis, we used the Bayesian Dirichlet equivalent (BDe) score (Heckerman et al., 1995). Assuming that the distribution of each node in the network can be learned independently of all other distributions in the network and assuming Dirichlet priors, the BDe score can be expressed as (Cooper and Herskovits, 1992; Heckerman, 1995)

(3) $$\begin{array}{l} \log\mathrm{Pr}(D|G)=\sum_{i,k}(\log\frac{\Gamma \left({\text{Z}}^{\prime }{}_{ik}\right)}{\Gamma \left({\text{Z}}^{\prime }{}_{ik}+{\text{Z}}_{ik}\right)}\\ \;+\sum_{j}\log\frac{\Gamma \left({\text{Z}}^{\prime }{}_{ijk}+{\text{Z}}_{ijk}\right)}{\Gamma \left({\text{Z}}^{\prime }{}_{ijk}\right)}) \end{array}$$

where Γ(*x*) is the Gamma function satisfying Γ(*x* + 1) = *x*Γ(*x*) and Γ(1) = 1, *Z_ik_* = ∑*_j_Z_ijk_*, *Z_ijk_* is the number of times variable *r^(t)^_i_* = *j* (where *j* is equal to “0” for no spike and “1” for a spike) and **r**_π(*i*)_(1 : *t*) = *k*, *Z*′_*ik*_ = ∑_*j*_*Z*′_*ijk*_ and *Z*′_*ijk*_ = *a* Pr(*r^(t)^_i_* = *j*, **r**_π(*i*)_(1 : *t*) = *k*|*G*_0_), where *a* is the equivalent sample size, *G*_0_ is a prior structure and π(*i*) is defined by the considered structure *G*. The search starts with an initial random structure. At each step, the network is modified by either adding a new connection, remove an existing one or reversing its direction. A search is then carried out through the space of all possible structures to find the model with the maximum score—or equivalently that best explains the observed data. In our implementation, we used the Bayesian Network Inference with Java Objects (BANJO) toolbox (Smith et al., 2006) with simulated annealing search algorithm (Kirkpatrick et al., 1983).

In a previous study, we examined the extent to which DBNs could infer the structure of a simulated network when neurons forming this network obey an inhomogeneous Poisson firing model (Eldawlatly et al., 2010), which results in significant variability in the temporal structure of the firing patterns, eventually mimicking what is typically observed in cortex (Shadlen and Newsome, 1998). Specifically, we examined the ability of DBN to identify the structure of a network when the following parameters were varied: synaptic delay, number of pre-synaptic connections, excitation-to-inhibition ratio, model memory, background firing rate, population size and analysis time window. We found that DBNs could faithfully reconstruct the network structure—even for relatively weak connectivity—within intervals (model memory) in the order of ~18 ms. This temporal resolution far supersedes other methods that have been typically used to infer connectivity from macroscale activity (such as functional Magnetic Resonance Imaging fMRI).

### Data analysis

For each neuron, the total number of evoked spikes in a given trial for each stimulated whisker was calculated as the difference between the total number of spikes fired by the neuron within the 100 ms-trial and the total number of spikes fired by the neuron in the 100 ms window preceding the trial. The mean first-spike latency of each neuron for a given whisker was computed as the average time taken by the neuron to fire the first spike post stimulus onset. To quantify the similarity in the response of individual neurons to deflection of the spared whiskers, we computed the difference in the evoked number of spikes as well as the difference in the first-spike latency of each neuron across the spared whiskers. The similarity was then computed as [1 - the normalized absolute difference] where the absolute difference for each neuron was normalized by the maximum absolute difference across all neurons.

To infer stimulus-specific networks, a total of 100 datasets, 18 s each, for each whisker were extracted from the recorded 900 trials/whisker in each of the control and plasticity data. Each dataset was formed by concatenating 180 trials that were randomly selected from a uniform distribution of the 900 trials. We analyzed the same type of stimulus-driven layer V barrel cortex data in previous experimental study using the same dataset length of 18 s for each dataset (Eldawlatly and Oweiss, 2011). In that study, the inferred networks were verified by examining the extent to which the inferred connections were consistent with individual neurons response properties. Therefore, we used the same dataset length here given the similarity in the analyzed data. The spike trains of each dataset were analyzed using DBN with Markov lags in the range [1, 5] bins ([1, 5] ms) which is within the range of delays of chemical synapses in layer V of the barrel cortex (averages ~2 ms) (Schubert et al., 2001; Sun et al., 2006). Whenever a connection was inferred at more than one Markov lag, only the largest lag was considered. For each cell, the maximum number of pre-synaptic cells (incoming edges) was set to 10 to conform to the 10% average connectivity ratio (given the electrode yield) we encountered in the barrel cortex (Feldmeyer, 2012). We conservatively set the search time to 30 min to ensure all connections are captured. Our previous analysis demonstrated that for neuronal populations of size 20–30 neurons (similar to the population sizes we recorded in this study), an algorithm search time of 5 min would be sufficient (Eldawlatly et al., 2010).

The similarity between the inferred networks was quantified as follows (Eldawlatly and Oweiss, 2011): we first represented each inferred network as an *n* × *n* binary adjacency matrix *A*, where *n* is the total number of neurons in the network. Each element *A*(*i, j*) takes the value “1” if there is a connection from neuron *i* to neuron *j* and “0” if there is no connection between the corresponding neurons. For a given population of *n* neurons, *K* deflected whiskers and *M* datasets per whisker, all the adjacency matrices of the inferred networks were vectorized and stacked together into one *KM* × *n*^2^ matrix. PCA was then applied to this matrix to extract significant features from the inferred networks by projecting the adjacency matrices into a *p*-dimension network space, where *p* ≤ *n*^2^, that accounts for most of the variance in the networks (Luo et al., 2003). The distance *D*(*A_l_, A_m_*) between a pair of adjacency matrices *A_l_* and *A_m_* was defined as

(4) $$D\left(A_{l},A_{m}\right)=||q_{l}-q_{m}||$$

where *q_l_* and *q_m_* are the projections of *A_l_* and *A_m_* in the *p*-dimension network space, respectively, and ||.|| is the Euclidean distance (*l_p_*-norm) between the two projections. The number of principal components used *p* was set to 2. The average distance between the networks inferred for a given pair of whiskers *w*_1_ and *w*_2_, *D*(*w*_1_, *w*_2_), was defined as

(5) $$\bar{D}\left(w_{1},w_{2}\right)=\frac{2}{M^{2}}\sum_{l}\sum_{m}D\left(A_{l}^{w_{1}},A_{m}^{w_{2}}\right)$$

Finally, the similarity between a pair of networks was computed as: 1—the normalized distance between the networks' projections in the feature space.

## Results

**Figure 1:**
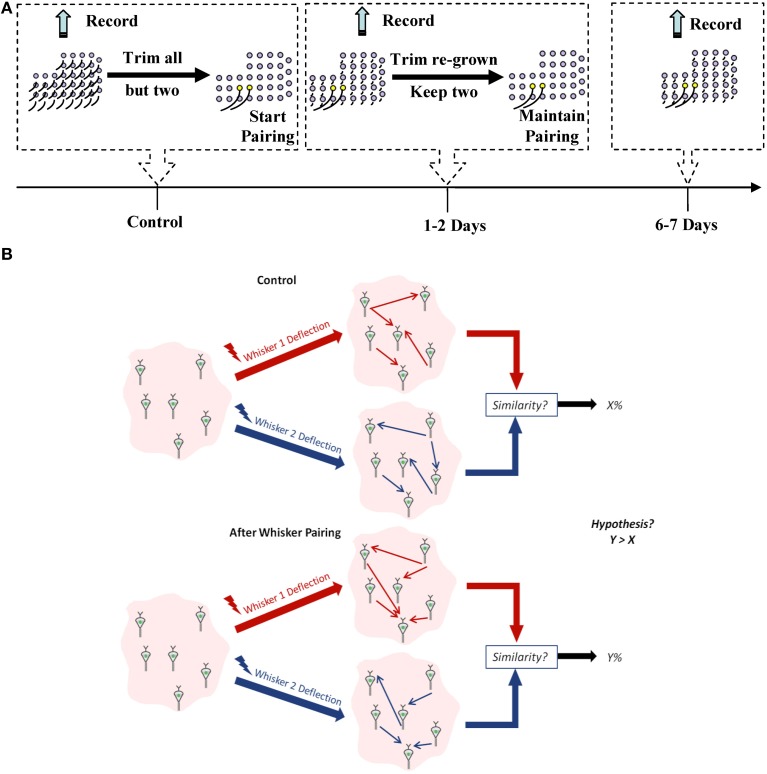
**Experimental design. (A)** Experiment timeline. Yellow follicles correspond to spared whiskers. **(B)** A schematic for our working hypothesis. Similarity between network representation of the spared whiskers' mechanical stimulation will increase when they are more frequently co-active compared to the control condition.

We chronically implanted each of the four rats with a 32-channel microelectrode array in vS1 and measured spiking activity from a total of 325 well-isolated single units in layer V in response to unilateral stimulation of individual whiskers. One week post-implantation, activity was recorded over a number of days (control data) after which whisker pairing on the contralateral side of the rat's mystacial pad was performed to induce plasticity. Activity was then recorded 1–2 days and 6–7 days post whisker pairing (plasticity data) (Figure 1A).

For each recorded ensemble, we used Dynamic Bayesian Networks (DBNs) to infer the effective connectivity between neurons in that ensemble (Eldawlatly et al., 2010; Eldawlatly and Oweiss, 2011). Using ideal observer analysis that has access to the distribution of the responses, we have previously demonstrated that DBNs provide more information about the deflected whisker identity than individual neurons' responses (Eldawlatly and Oweiss, 2011). This information represented the ordered, spike-by-spike, causal influence between neurons in the ensemble. We hypothesized that:

1. whisker pairing will increase the likelihood that co-active inputs from the spared whiskers reach the barrel cortex within a window of time that is shorter than if these whiskers were a subset of an intact whisker set.
2. cortical neurons in barrels innervated by the sparred whiskers will likely fire in a quasi–synchronous fashion in response to these near-simultaneous inputs, thereby promoting Hebbian-like plasticity (Feldman and Brecht, 2005).

We therefore expected that the co-activation of neurons in the spared-whisker barrels would induce STDP, eventually leading to increased similarity in the effective connectivity between these neurons (as illustrated by Figure 1B). For comparison, we computed the spike count and first-spike latency for each neuron in response to the deflection of each of the spared whiskers before and after pairing.

**Figure 2:**
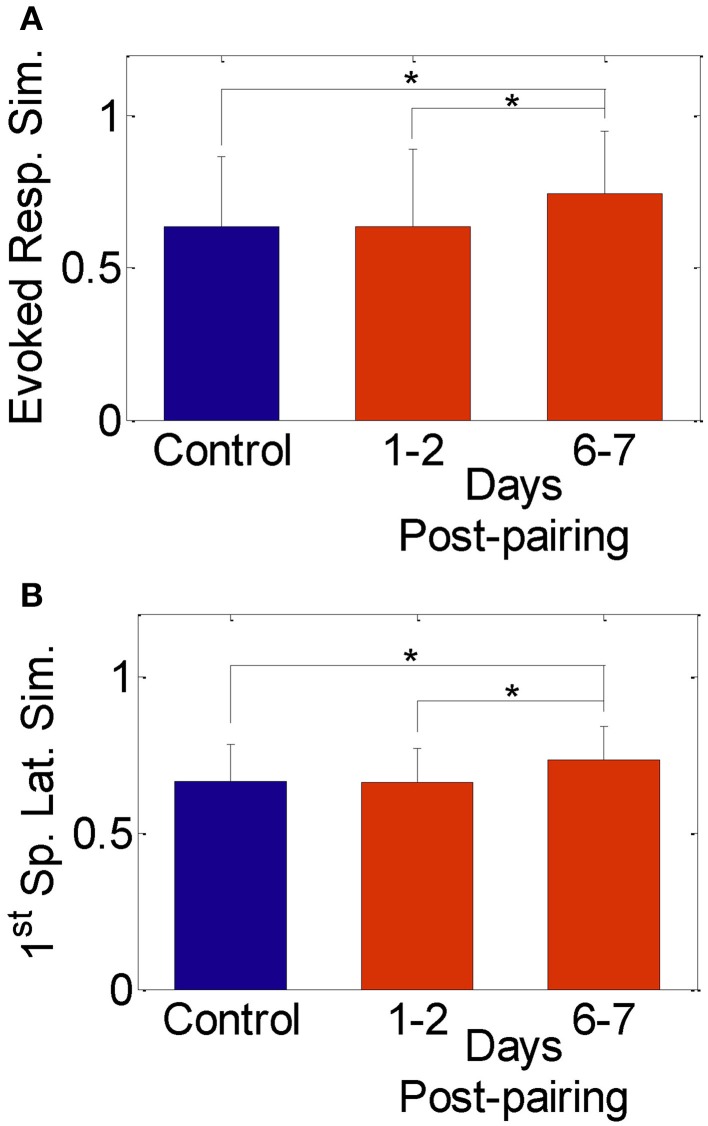
**Similarity between evoked responses in the spared-whisker barrels, averaged across neurons and subjects. (A)** 1–2 days and **(B)** 6–7 days post whisker-pairing (mean ± s.d.). Y-axis was computed as [1—the normalized absolute difference in evoked response in **(A)** and in first-spike latency in **(B)** of the spared whiskers] for each neuron (^*^P < 0.05, two-sample *t*-test). Responses of a total of 481 neurons recorded from four subjects were analyzed.

We found no significant change in the spike count and first spike latency 1–2 days post-pairing, similar to published reports that used similar single neuron measures (Diamond et al., 1993, 1994; Armstrong-James et al., 1994; Lebedev et al., 2000). The similarity between responses to stimulation of the spared whisker pair, however, increased significantly 6–7 days post-pairing (Figures 2A,B; Normalized similarity in evoked spikes for control: 0.64 ± 0.23, 6–7 days post-pairing: 0.74 ± 0.21; Normalized similarity in first-spike latency for control: 0.66 ± 0.12, 6–7 days post-pairing: 0.73 ± 0.11, *P* < 0.05, two-sample *t*-test). Thus, our results replicate those in published reports confirming that pairing-induced changes in cell excitability become observable beyond the 2-day window post-pairing.

**Figure 3:**
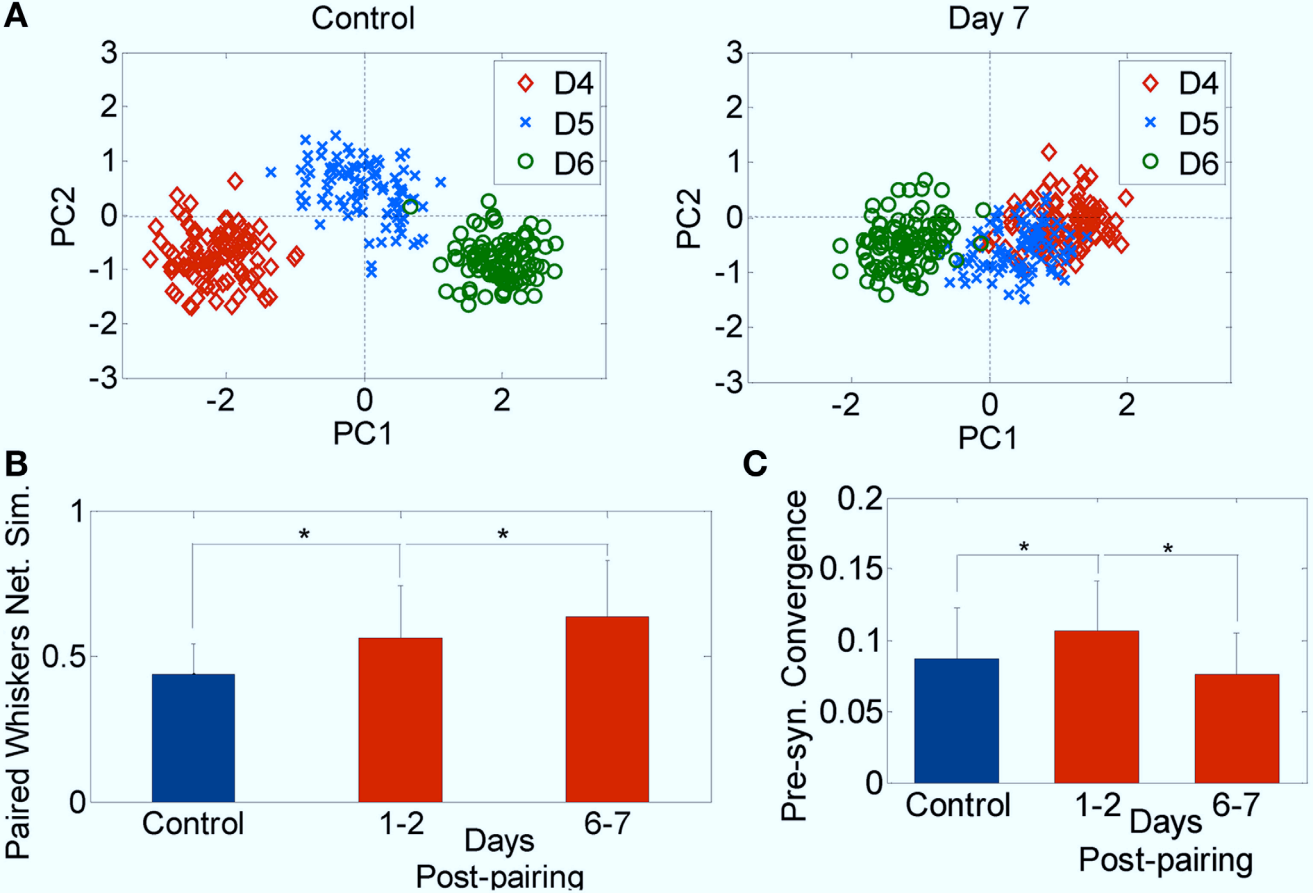
**Network analysis. (A)** Network feature space of a sample rat for 3 whiskers (D4, D5, and D6) for the Control (left) and 7-days post D4–D5 whisker pairing (right). Each dot corresponds to the projection of one network onto a 2-dimension principal component feature space. **(B)** Network similarity across spared whiskers for control (blue) and plasticity data recorded 1–2 days and 6–7 days post-pairing (red) averaged across 4 subjects (mean ± s.d.). **(C)** Pre-synaptic convergence averaged across neurons for control (blue) and plasticity data (red) (mean ± s.d.). ^*^P < 0.05, two-sample *t*-test.

The effective connectivity between neurons in both control and plasticity data was computed and used to construct a network feature space as described in the materials and methods section (Eldawlatly et al., 2010; Eldawlatly and Oweiss, 2011). In this space, ensemble responses appear as a cluster of points where each cluster corresponds to a specific input (in our case a specific whisker deflection). Similarity between networks corresponding to different inputs would be manifested in the network output space as a *cluster merging* process. Figure 3A illustrates the network feature space of control data and 7 days post-pairing. Each point represents the projection of a network corresponding to a single 18-s long dataset (as explained in section Data Analysis) comprising the response to deflection of one of the three whiskers D4, D5, and D6. The figure demonstrates the merging between D4 and D5 clusters in one rat after pairing these two whiskers, suggesting that the network response in the D4 barrel and that in the D5 barrel became increasingly similar, while remained dissimilar for whisker D6 that was not part of the pairing process. Figure 3B summarizes the average similarity across all rats. The significant increase in similarity is proportional to the number of days post-pairing (Network similarity for control: 0.58 ± 0.1, 1–2 days post-pairing: 0.66 ± 0.1, 6–7 days post-pairing: 0.71 ± 0.1, *P* < 0.05, two-sample *t*-test). Thus, the ensemble response to a spared whisker stimulation became increasingly similar to that of the other spared whisker. This suggests that re-organization of the receptive fields of the neurons in one spared-whisker barrel engulfs inputs that would normally trigger the strongest response in the other spared-whisker barrel. A significant change in the effective connectivity was observed 1–2 days post-pairing, a phenomenon not observed when analyzing individual neuron firing properties during the same period.

We also found an early increase followed by a late decrease in pre-synaptic convergence—the number of pre-synaptic inputs averaged across neurons and subjects—(Control: 0.09 ± 0.04, 1–2 days post-pairing: 0.12 ± 0.04, 6–7 days post-pairing: 0.08 ± 0.03, *P* < 0.05, two-sample *t*-test; Figure 3C). The early increase suggests that long-term potentiation (LTP) might have dominated, while the late decrease suggests the possible occurrence of long term depression (LTD) (Feldman et al., 2004). Given the large extent of convergence of functionally independent inputs from other cortical laminae onto infragranular layer V neurons, the rapid changes detected in our data after 1–2 days post-pairing suggest that sensory map re-organization could be mediated by spike timing-dependent plasticity (STDP), and in particular LTP and LTD of existing connectivity, while less likely by dendritic mechanisms which require at least 2–4 days up to a month to contribute to the induced plasticity (Trachtenberg et al., 2002).

**Figure 4:**
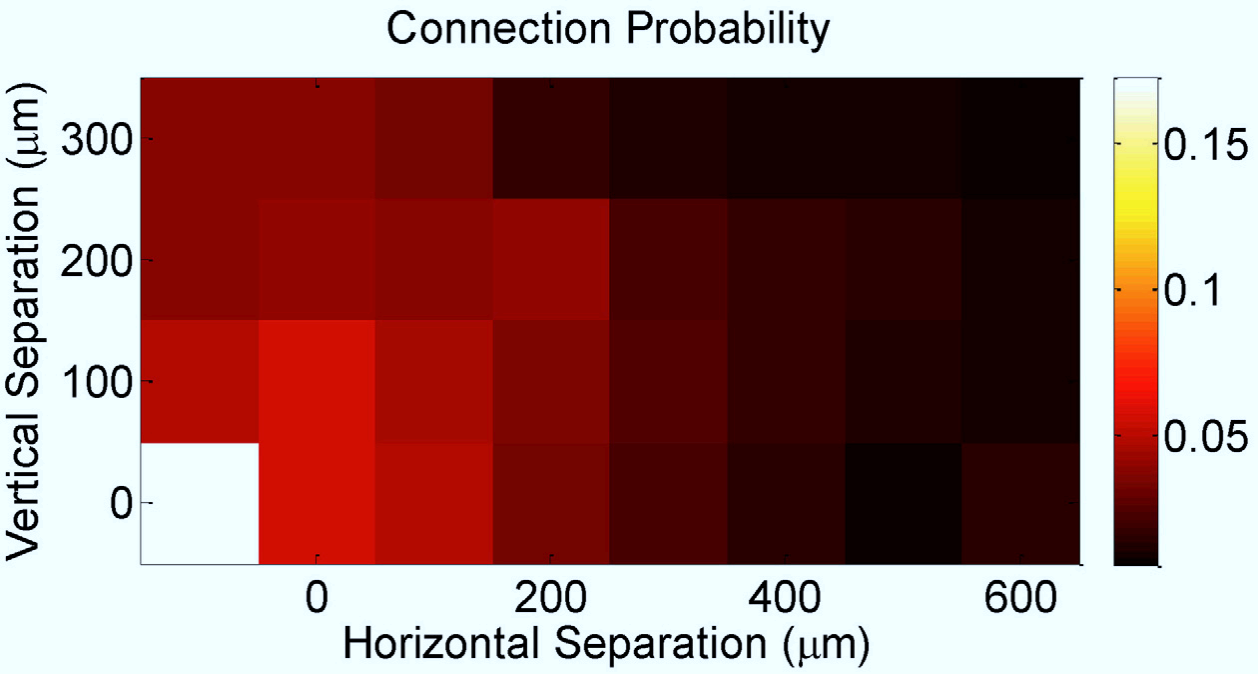
**Effective connection probability as a function of the horizontal and vertical separations between the electrodes on which neurons were recorded**.

Finally, DBN analysis allowed us to examine *the extent of convergence* in the circuit, irrespective of whether the inferred connectivity is an actual indicator of actual synaptic connectivity or not. In particular, it has been suggested that the longitudinal effects of pre-synaptic convergence on the magnitude and type of plasticity depends on the number of converging synapses and their strength (Feldman et al., 2004). Consistent with previous studies using patch-clamp recordings of postsynaptic potentials (PSPs) confirming that synaptic connectivity in cortex is predominantly *local* (Petersen et al., 2001, 2002, 2003; Feldman and Brecht, 2005; Song et al., 2005; Hofer et al., 2011; Ko et al., 2011), our network-based metric of effective connectivity demonstrated strong evidence in support of these studies, as measured by an increasing probability of graph connectedness between a given pair of neurons with decreasing vertical and horizontal separation between electrodes that recorded the pairwise activity (Figure 4).

## Discussion

Historically, quantifying plastic changes in neural connectivity has been primarily confined to two domains of analysis: (1) the synaptic level, owing largely to the feasibility of measuring changes in postsynaptic potentials under current clamp in pairs or triplets of neurons *in vitro* and more recently *in vivo* (Kodandaramaiah et al., 2012) and (2) the voxel level, owing to the feasibility of performing functional brain imaging experiments *in vivo*, for example, using fMRI. In between, quantifying plasticity-induced changes in connectivity at the population level has been significantly lagging. Herein, we provided a novel way to detect changes in population dynamics that could not be detected when analyzing single neuron response properties. In particular, the changes in the functional connectivity between neurons in layer V as a result of whisker pairing suggested by our data imply that large scale sensory map re-organization occurs much more rapidly than previously thought, and that it is consistent with an STDP rule. This mechanism has been implicated in a wide range of studies that examined LTP and LTD of synapses following tetanic stimulation of pre- and post-synaptic cells (Bi and Poo, 1999). Furthermore, STDP has been proposed in multiple studies as a potential mechanism that mediate changes in neuronal responses as a result of whisker pairing (Feldman, 2000; Rema et al., 2006; Jacob et al., 2007), but evidence that support the time course of these changes has been lacking. Because Dynamic Bayesian Network (DBN) is sensitive to temporal resolutions of the order of milliseconds (Eldawlatly et al., 2010), it is readily capable of detecting changes occurring within the STDP time-scale.

It is plausible that rats may alter their whisking behavior following whisker trimming in ways that could reduce the concurrent activation of spared-whisker barrels, and thus reduce the likelihood of engaging an STDP mechanism. Studies of whisking behavior, however, have shown that rats move their whiskers in a repetitive retraction-protraction cycle—often referred to as *whisking bout*—to palpate external objects. This repetitive motion may involve multiple whiskers following similar bouts such as whisking in air, or could involve markedly different self-generated patterns, such as when whiskers strike the surface of an object. Trimming of some whiskers implies that a substantial portion of the input to the barrel cortex has been lost. It is currently unknown if animals compensate for this lost input by altering the self-generated movement patterns of the spared whiskers in order to gather a qualitatively similar amount of information about the touched object compared to when they use the same whiskers as part of an intact whisker set. Rats may choose to move spared whiskers in a more coordinated fashion in order to make rapid decisions about sensory stimuli, as has been demonstrated by studies that examined the extent to which spatially distributed sensory inputs affects speed and accuracy of decision making during simple sensory detection, albeit at the single whisker level (Celikel and Sakmann, 2007). Co-active inputs, while may provide temporally redundant sensory information to the barrel cortex, can nevertheless serve to increase information about the surrounding when integrated with spatial information. The adjacency of the spared whiskers may contribute to this increased likelihood. One limitation of our current study though is that we have not carried out independent measurements of whisking behavior to provide support for this idea, largely because it is an insurmountable challenge to measure the volitionally controlled whisker movements in a freely behaving animal at the spatial and temporal resolution needed to address this question. Nonetheless, our overarching hypothesis is that whisker pairing increases the likelihood that co-active inputs from the spared whiskers reach the barrel cortex within interval lengths similar to the case when the entire whisker set is intact. Therefore, cortical neurons in barrels innervated by the spared whiskers will likely fire in a quasi–synchronous fashion in response to these near-simultaneous inputs, thereby promoting STDP (Feldman and Brecht, 2005).

There is an abundance of published reports demonstrating that whisker pairing induces experience-dependent plasticity as early as the first few hours of pairing in layer IV (Diamond et al., 1993; Rema et al., 2006; Quairiaux et al., 2007; Sellien and Ebner, 2007). Our present study suggests that these changes may well extend to layer V over a similar time scale, perhaps owing to the existence of excitatory across-layer connectivity (Feldmeyer, 2012). On the other hand, *in vivo* imaging of dendritic structures in layer V provides evidence that sensory deprivation induces changes in the turnover of spines starting as early as 1–2 days following deprivation (Trachtenberg et al., 2002). This suggests that experience-dependent plasticity may be manifested as early as 1–2 days post-pairing. However, these changes may be too subtle to be detected at the *individual neuron* level, but that network analysis that accounts for temporal precision of spiking can reveal such changes.

Compared to previous studies of experience-dependent plasticity in the rat barrel cortex following sensory deprivation, our experimental paradigm provides a more accurate account of the effects induced by sensory deprivation for two reasons: First, our study was designed to permit identifying plastic changes by recording evoked responses to whisker deflection *in the same subject* using a chronically implanted electrode array over a number of days. This enabled us to determine the extent of response variability in control and experimental conditions more accurately than if it were to be assessed across different subjects. Previous studies, on the other hand, acutely recorded neural responses to whisker deflection and compared them across *different subjects* (Diamond et al., 1993, 1994; Armstrong-James et al., 1994; Lebedev et al., 2000). We believe that the approach in these studies might have been susceptible to across-subject variability that could not be fully attributed to short-term plasticity effects. In addition, very few studies have examined plasticity in vSI Layer V. As this layer consists the major output of the barrel system and heavily innervates vibrissal primary motor cortex (vMI) (Hooks et al., 2011), characterizing the neural substrate that mediates the changes during whisking behavior is critical. Our results are consistent with other reports (Jacob et al., 2012) that documented the occurrence of rapid plasticity in layer V following sensory deprivation. Jacobs et al.'s assessment of plasticity, however, was conducted across different groups of animals and over a longer time interval (Control, 3-day and 10-day deprivation groups). Our study, on the other hand, fills a knowledge gap about the extent of re-organization in local cortical circuits *in the same animal* over a shorter interval.

Second, with the exception of only one study that quantified changes in the cross-correlograms of pairs of neurons in the barrels of the spared whiskers (Lebedev et al., 2000), our study is the first, to our knowledge, that uses a novel quantitative way to track whisker pairing-induced plasticity at the ensemble level. Single-neuron response properties, namely spike count and precise spike timing, have been traditionally used as the sole neuronal response properties for assessing plasticity-induced changes in receptive field characteristics. Our previous study (Eldawlatly and Oweiss, 2011), however, demonstrated that these two metrics are more susceptible to intrinsic, across-trial variability in single neuron responses in vS1. In addition, the ensemble response property—measured by the functional connectivity metric derived from DBN analysis—was shown to be more informative about stimulus identity than any of the single-neuron response properties we assessed. Because it is a population metric, it lends itself naturally to the assessment of plasticity-induced changes within local circuits that can be tracked over short periods within the same subject. This also explains why this metric could detect significant plasticity-induced changes within the first 48 h than single-neuron metrics. Our experimental design, however, could not independently verify whether the observed changes in the effective connectivity over longer periods resulted from the emergence of new connections across the sparred-whisker barrels or due to other non-Hebbian forms of plasticity, as suggested by previous reports (Fox, 2002; Feldman and Brecht, 2005).

The observed increase in pre-synaptic convergence 1–2 days post whisker pairing suggested by our data implies an overall increase in the number of pre-before-post spiking events, indicating that LTP could have possibly been dominating. On the other hand, the drop in convergence to postsynaptic cells suggested by our data 6–7 days post whisker pairing suggests that synapses were strengthened enough for the corresponding changes in spiking patterns to be captured by the DBN, perhaps as a result of correlated inputs to these neurons, and that LTD is more likely to have occurred. In each of these cases, the proportion of LTP vs. LTD depends on the degree of correlation in the sensory inputs that the pre-synaptic neurons receive. Because whisker pairing may increase the likelihood of timing correlation between inputs to sparred-whisker barrels, our findings support the hypothesis that timing-dependent plasticity contributes to cortical re-organization following sensory deprivation. Nonetheless, other mechanisms that regulate network excitability through synaptic scaling to prevent unconstrained potentiation in order to reach homeostatic stable network states could also be at play (Turrigiano and Nelson, 2000; Turrigiano, 2008), and further studies are certainly needed to elucidate the temporal and spatial scales of these mechanisms.

## Author contributions

Seif Eldawlatly and Karim G. Oweiss designed research. Seif Eldawlatly performed research and analyzed data. Seif Eldawlatly and Karim G. Oweiss wrote the manuscript.

### Conflict of interest statement

The authors declare that the research was conducted in the absence of any commercial or financial relationships that could be construed as a potential conflict of interest.
